# Non-invasive programmed stimulation to identify high-risk patients with implanted cardioverter defibrillator (the NIPS-ICD study): study protocol for a randomized controlled trial

**DOI:** 10.1186/s13063-016-1170-2

**Published:** 2016-01-27

**Authors:** Piotr Futyma, Marian Futyma, Piotr Kułakowski

**Affiliations:** Invasive Cardiology Department, St. Joseph’s Heart Centre, Anny Jagiellonki 17, 35-623 Rzeszów, Poland; Postgraduate Medical School, Grochowski Hospital, Grenadierów 51/59, 04-073 Warsaw, Poland

**Keywords:** Implantable cardioverter defibrillator, noninvasive programmed stimulation, risk stratification

## Abstract

**Background:**

The use of an implantable cardioverter defibrillator (ICD) is a widely used and effective therapy, which reduces the risk of cardiac death in many cardiac diseases, both implanted for secondary and primary prevention. However, recurrent arrhythmias and ICD discharges have adverse prognostic significance. Additional parameters that would identify patients who are at increased risk of arrhythmias and appropriate ICD interventions would be of clinical value. Modern ICDs are relatively complex devices with a number of functions, including the possibility to perform noninvasive programmed stimulation (NIPS) with an implanted electrode located in the right ventricle.

**Methods/Design:**

The aim of the study is to evaluate the usefulness of NIPS in determining the likelihood of life-threatening arrhythmic events in patients with ICD. The study will include 150 consecutive patients with an ICD implanted both for primary and secondary prevention, regardless of etiology, who are followed in the outpatient clinic of our center and do not meet the exclusion criteria. A 12-step St. George’s Hospital NIPS protocol using ICD will be performed. The endpoint is to induce sustained ventricular arrhythmia (VT lasting more than 30 seconds or hemodynamically unstable VT/VF) or the end of the protocol. In case of serious and/or hemodynamically unstable heart rhythm disorders resistant to treatment with a low-energy antiarrhythmic pacing (ATP), the patient receives a short-term intravenous general anesthesia, and internal or external defibrillation is performed. Outpatient follow-up will be conducted during the pre-scheduled ICD control visits. An analysis of records of a registered memory device will be collected, a patient will be interviewed, and physical examination will be carried out. The follow-ups will be held every 3 months for 1 year. The primary endpoint of the follow-up will be appropriate intervention of ICD or sudden cardiac (arrhythmic) death; the secondary, appropriate ICD intervention, or death from cardiovascular causes; and the tertiary, appropriate ICD intervention, death or hospitalization for cardiovascular causes.

**Discussion:**

It is expected that appropriate ICD interventions during follow-up will occur more often in patients who had sustained ventricular arrhythmias induced during NIPS.

**Clinical trials registry:**

ClinicalTrials.gov, NCT02373306, date of registration: 26 February 2015.

## Background

Implantable cardioverter defibrillator (ICD) is a widely used and effective therapy that reduces the risk of cardiac death in many cardiac diseases, and is implanted for secondary and primary prevention [1–9]. In the case of new-onset or recurrence of life-threatening ventricular arrhythmia, the device interrupts it by anti-arrhythmic pacing (ATP) or by electrical shock. It is known that recurrent arrhythmias and ICD discharges have adverse prognostic significance. ICD therapeutic interventions are often associated with the sensation of pain, reduce the device’s battery life, are associated with the risk of serious proarrhythmia, and can shorten the patient’s life [10–13]. Moreover, in the case of hemodynamically unstable arrhythmia, the late arrival of the device’s intervention may not protect patients from syncope and their consequences [14].

In view of the above-mentioned aspects, additional parameters that would identify patients who are at increased risk of arrhythmias and appropriate ICD interventions would be of clinical value. Some studies have shown that factors predicting ventricular tachycardia (VT) or ventricular fibrillation (VF) recurrences, and consequently ICD interventions, include the patient’s age, NYHA class heart failure, left ventricular ejection fraction (LVEF), the presence of atrial fibrillation (AF), the QRS complex width, and the status of renal function [15, 16]. However, the accuracy of these parameters is limited, and other variables that may identify vulnerable patients are sought. This may have important clinical implications because such patients could receive more aggressive antiarrhythmic therapy or may undergo prophylactic ablation of arrhythmia substrate to prevent the occurrence of arrhythmias and ICD discharges. Indeed, some studies have suggested that it might be worthwhile to perform prophylactic ablation of VT in patients with previously implanted ICD [17, 18]; however, this approach has not entered into daily clinical practice [19].

One of the methods of risk stratification for sudden cardiac death that has been used for many years is programmed ventricular stimulation (PVS) [20, 21]. This is an invasive study assessing the likelihood of VT or VF induced by stimulation to occur. This test also has been used in order to qualify for prophylactic ICD implantation in patients with LVEF 31 to 40 % and nonsustained VT in ambulatory ECG [1, 9]. The primary disadvantage of PVS is its invasiveness. Modern ICDs are relatively complex devices with a number of functions, including the possibility to perform non-invasive programmed stimulation (NIPS) with an implanted electrode located in the right ventricle. NIPS is readily available, inexpensive, minimally burdening the ICD battery, and less disturbing to the patient, because it does not require any invasive procedure [22–24].

Some studies suggested that appropriate interventions occur more frequently in patients who had ventricular arrhythmias induced by NIPS, but the prognostic significance of NIPS has not yet been determined [22, 25, 26]. One of the first NIPS-related studies demonstrated that VF/VT inducibility during the test of the device can help to optimize the ICD programming; however, the predictive value of NIPS had not been studied at that time [22]. In another study, one of the first and few on the prognostic value of NIPS, it has been shown that induction of monomorphic, especially relatively slow VT (cycle length > 280 ms) was prognostic for recurrence of arrhythmias [25]. This study was relatively small, the distribution of the etiology had not been considered, and some of the results were surprising; for example, previous myocardial infarction of inferior wall, rather than the anterior wall, predicted altered outcome, whereas the LVEF had no prognostic value. In addition, these studies were conducted many years ago, when the use of primary angioplasty for acute myocardial infarction was low, and therefore the clinical characteristics of the patient groups from that period are different than those of contemporary patients.

## Methods/Design

The study protocol was approved by the Ethics Committee of Regional Medical Chamber in Rzeszów and is in full compliance with the Declaration of Helsinki. Approval Number: 77/2013/B.

### The aim of the study

The aim of the study is to evaluate the usefulness of NIPS in determining the likelihood of life-threatening arrhythmic events in patients with ICD.

### Methodology/Design

#### The study group

The study will include 150 consecutive patients with an ICD implanted both for primary and secondary prevention, regardless of etiology, who are followed in the outpatient clinic of our center and do not meet the following exclusion criteria:

1. lack of consent for NIPS
2. decompensated heart failure
3. unstable angina
4. persistent/long standing AF without effective anticoagulation (risk of sinus rhythm return during NIPS)
5. thrombus in the left ventricle
6. appropriate device interventions during the 40 days prior to planned NIPS
7. pacing/sensing problems

After selecting a patient to undergo NIPS, the following parameters will be analyzed: etiology, age, sex, and anthropometric measurements (weight, height, and waist), echocardiogram performed within 3 months after enrollment (including LVEF and end-diastolic dimension of LV), NYHA class, presence of AF, state of renal function, width of native and paced QRS complex, occurrence of ventricular arrhythmias (detected on ECG or EGM), previously performed cardiac surgery and/or coronary angioplasty, occurrence of ICD interventions in the past, and current drug therapy.

#### NIPS methodology

On admission, the patient will be fasting. After interviewing and physical examination, the patient will be informed about the manner and purpose of the NIPS, and written consent will be obtained. The ECG will be recorded, and access to the venous system will be obtained. The patient will be transferred to the EP room. Throughout the duration of the NIPS, surface 12-lead ECG from an electrophysiological system will be recorded. ICD will be interrogated, and a review of the available events and interventions in memory ICD will be performed. The parameters of the detection of VT/VF and therapy parameters will be set out, as well as the parameters of stimulation and sensing: the low and high voltage impedance, the signal from the defibrillation lead, and the pacing threshold.

Next, a 12-step St. George’s Hospital NIPS protocol using ICD will be performed [27, 28], which consists of introduction of a single and double extrastimuli from the pacing lead during sinus rhythm (or atrial fibrillation if present) and after eight-beat extrastimulus drive trains at paced cycle lengths of 600, 500, and 400 ms. If a sustained arrhythmia will not be inducible, the procedure will be repeated using triple extrastimuli, wherein in step 12 the three premature impulses after the eight-beat drive at cycle length of 400 ms are delivered.

The endpoint is to induce sustained ventricular arrhythmia (VT lasting more than 30 seconds or hemodynamically unstable VT/VF) or the end of the protocol. In case of ventricular arrhythmias induction, the following parameters will be defined:

1. arrhythmia-induction protocol (stage of NIPS at which arrhythmia was induced)
2. the type of arrhythmia (single extrasystoles, couplets, nonsustained VT (nsVT), sustained VT (sVT), or VF)
3. In the case of VT/VF occurrence:

- mean tachycardia cycle length calculated from the first 10 QRS complexes
- effectiveness of ATP in terminating arrhythmia
- in the case of ineffectiveness of ATP - efficacy of internal cardioversion/defibrillation.

In case of serious and/or hemodynamically unstable heart rhythm disorders resistant to treatment with a low-energy ATP, the patient receives a short-term intravenous general anesthesia, and internal or external defibrillation is performed.

If the test induces sustained ventricular arrhythmia, after the sinus rhythm has been restored, the patient will be transferred to the intensive care room, where at the time of his stay, continuous ECG and blood pressure will be performed.

#### Outpatient follow-up

The follow-up will be conducted at the outpatient clinic for patients with ICD located in our center. Analysis of records of registered memory device classified as VT/VF episodes will be collected in printed and electronic versions for further investigation by the study team. Medical history will be taken, and a physical examination will be carried out. In case of endpoint VT/VF occurrence during follow-up, the time from NIPS test to ICD episode detection/therapy will be measured. The follow-up will last one year and will be conducted every 3 months; thus, each patient will have four visits – one at 3, 6, 9 and 12 months after performing NIPS. The following parameters will be collected:

1. The patient’s clinical status will be determined on the basis of a physical examination, NYHA class, changes in medication, hospitalizations, cardiac events such as syncope, ICD discharges, palpitations and other, which occurred after the previous visit.
2. The ICD memory will be examined, and all events will be analyzed. All ICD interventions will be analyzed and classified as appropriate or inappropriate. The type of cardiac rhythm (VF, VT, or other) and the cycle length and duration will be also measured.
3. All follow-up visits and data collection will be performed by the principal investigator (PF). If necessary, drug therapy will be modified during follow-up, but the result of the NIPS will not be taken into account when changes in the antiarrhythmic drug therapy are considered. Changes in ICD programming will be possible; however, these will be based on arrhythmic events occurring during follow-up and not based on the results of the NIPS.

### Expected results

During the NIPS the following clinical situations are expected to occur:

1. lack of induction of arrhythmia (negative result)
2. induction of ventricular extrasystoles (negative result)
3. induction of hemodynamically stable nsVT (negative result)
4. induction of hemodynamically unstable nsVT (positive result)
5. sVT induction (positive result)
6. VF induction (positive result).

It is expected that appropriate ICD interventions during follow-up will occur more often in patients who had sustained ventricular arrhythmias induced during NIPS (positive result). The primary endpoint of the follow-up will be appropriate intervention of ICD or sudden cardiac (arrhythmic) death; the secondary, appropriate ICD intervention or death from cardiovascular causes; and the tertiary, appropriate ICD intervention, death or hospitalization for cardiovascular causes. Because all patients will remain under the study follow-up for 1 year, the degree of exposure to arrhythmia recurrence (arrhythmia burden) will be determined defined as the number of relevant interventions during the year of observation.

Appropriate ICD intervention is defined as the high or low energy treatment due to the occurrence and detection of VT/VF.

For death from cardiovascular causes, it is considered death due to VF, extensive myocardial infarction, end-stage heart failure, or electromechanical dissociation or asystole due to presumed cardiovascular cause.

For sudden cardiac (arrhythmic) death, death due to sVT/VF or, in the absence of available documentation, death that occurred within 1 hour of the onset of symptoms [29] may be considered. Every effort will be made to interrogate ICD postmortem in order to establish the mode of death.

### Statistical methods

The principal analysis will examine the relationship between positive NIPS result and the follow-up outcome. Further subanalyses will also take into account the protocol stage at which arrhythmia was induced as well as the tachycardia cycle length and duration. At the first stage, groups of patients will be extracted by the occurrence of arrhythmic events during NIPS,- as in section 4. Afterwards, these groups will be compared according to the data collected during the follow-up (endpoints). The group, in which the endpoints occurred, will be compared with a group of patients without endpoints according to the NIPS result using Kaplan–Meier survival curves and tested with a log-rank test. A P value of 0.05 will be considered statistically significant for all analyses.

Next, with Multivariate Cox Proportional Hazards Model the presence or absence of the NIPS result correlation will be determined with other risk factors affecting the possibility of developing adequate ICD interventions that have been previously described in the available literature and widely accepted, that is, echocardiogram result, NYHA class, the presence of atrial fibrillation, renal function, width of native QRS complexes. The sensitivity, specificity, positive, and negative predictive value in predicting the occurrence of NIPS endpoints also will be calculated using Student’s t test or the Mann–Whitney test for continuous variables, where appropriate.

With the chi-square test, 150 patients have been estimated as needed for inclusion in the study. This assumption is based on the calculation that primary endpoints will occur in 6.7 % patients, and the result of NIPS will be abnormal in 30 % of those patients and in 7.9 % of patients without VT/VF recurrences [2, 30, 31]. This number of patients will be sufficient to demonstrate significant difference (*P* < 0.05) between the groups.

## Discussion

This study will provide information regarding the result of NIPS on the occurrence of life-threatening arrhythmic events in patients with ICD. It is expected that appropriate ICD interventions during follow-up will occur more often in patients who had sustained ventricular arrhythmias induced during NIPS. Consequently, these patients may require more aggressive antiarrhythmic therapy or prophylactic ablation of arrhythmia substrate to prevent the occurrence of arrhythmias and ICD discharges.

### Limitations

The study has some limitations. First, although all consecutive patients attending our outpatient clinic will be invited to the study, probably not all will give consent to enter the trial. Thus, the study group will consist of all consecutive patients who agreed to participate in the study but not of all our patients with ICD. However, a logbook including all ICD patients will be maintained, so we will know exactly what percentage of our ICD patients entered the study. Secondly, the physician performing follow-up visits will not be blinded to the results of NIPS. However, as a rule, medication or ICD programming will not be influenced by NIPS results because there are no data indicating that this should be done. It is our and similar studies which should shed more light on this issue and answer the question whether NIPS results can be taken into account when medication and ICD programming changes are concerned.

### Trial status

This study is currently recruiting participants.

## Abbreviations

AF: atrial fibrillation
EP: electrophysiology
ICD: implantable cardioverter defibrillator
LVEF: left ventricular ejection fraction
NIPS: non-invasive programmed stimulation
nsVT: nonsustained VT
NYHA: New York Heart Association
PVS: programmed ventricular stimulation
sVT: sustained VT
VF: ventricular fibrillation
VT: ventricular tachycardia.
